# Comprehensive copy number profiles of breast cancer cell model genomes

**DOI:** 10.1186/bcr1370

**Published:** 2006-01-03

**Authors:** Ashleen Shadeo, Wan L Lam

**Affiliations:** 1 Cancer Genetics and Developmental Biology, British Columbia Cancer Research Centre, Vancouver BC, V5Z 1L3, Canada

## Abstract

**Introduction:**

Breast cancer is the most commonly diagnosed cancer in women worldwide and consequently has been extensively investigated in terms of histopathology, immunochemistry and familial history. Advances in genome-wide approaches have contributed to molecular classification with respect to genomic changes and their subsequent effects on gene expression. Cell lines have provided a renewable resource that is readily used as model systems for breast cancer cell biology. A thorough characterization of their genomes to identify regions of segmental DNA loss (potential tumor-suppressor-containing loci) and gain (potential oncogenic loci) would greatly facilitate the interpretation of biological data derived from such cells. In this study we characterized the genomes of seven of the most commonly used breast cancer model cell lines at unprecedented resolution using a newly developed whole-genome tiling path genomic DNA array.

**Methods:**

Breast cancer model cell lines MCF-7, BT-474, MDA-MB-231, T47D, SK-BR-3, UACC-893 and ZR-75-30 were investigated for genomic alterations with the submegabase-resolution tiling array (SMRT) array comparative genomic hybridization (CGH) platform. SMRT array CGH provides tiling coverage of the human genome permitting break-point detection at about 80 kilobases resolution. Two novel discrete alterations identified by array CGH were verified by fluorescence *in situ *hybridization.

**Results:**

Whole-genome tiling path array CGH analysis identified novel high-level alterations and fine-mapped previously reported regions yielding candidate genes. In brief, 75 high-level gains and 48 losses were observed and their respective boundaries were documented. Complex alterations involving multiple levels of change were observed on chromosome arms 1p, 8q, 9p, 11q, 15q, 17q and 20q. Furthermore, alignment of whole-genome profiles enabled simultaneous assessment of copy number status of multiple components of the same biological pathway. Investigation of about 60 loci containing genes associated with the epidermal growth factor family (epidermal growth factor receptor, HER2, HER3 and HER4) revealed that all seven cell lines harbor copy number changes to multiple genes in these pathways.

**Conclusion:**

The intrinsic genetic differences between these cell lines will influence their biologic and pharmacologic response as an experimental model. Knowledge of segmental changes in these genomes deduced from our study will facilitate the interpretation of biological data derived from such cells.

## Introduction

Breast cancer is the most prevalent cancer worldwide and is the second leading cause of cancer-related deaths in women in North America [1,2]. It is a complex disease in which multiple genetic factors can combine to drive pathogenesis [3-5]. Changes in copy numbers of genes such as *ERBB2 *and c-*MYC *have been extensively documented in breast cancer and are present in model cell lines [6-9]. Amplified (and overexpressed) genes are prime therapeutic targets as for example, the use of the drug trastuzumab against ERBB2 has been shown to improve breast cancer survival rates alone or in combination with other treatments [10-12].

Strategies to detect gene copy number alterations will facilitate the identification of novel molecular targets. Previous studies with 10-megabase (Mb) resolution conventional metaphase comparative genomic hybridization (CGH) have identified gross regions of recurrent chromosomal aberrations in multiple breast cancer cell lines including loci within chromosomes 1q, 8q, 11q13, 17q and 20q13. Many of these alterations proved to be relevant because they were also present in primary tumors investigated [13-15]. Recent advances in array CGH have greatly improved the resolution of this technology, enabling the detection of segmental copy losses and gains [16,17]. Regional genomic arrays, providing contiguous or tiling coverage of a locus of interest, have been constructed for the fine mapping of commonly altered regions in breast cancer (such as 20q13) [18-20]. Whole chromosome arrays have been used to provide information at 500 kb intervals. For example, a chromosome 17 array was used to identify 13 regions of change present in breast cancer cell line models and primary breast cancers [21]. Similarly, a genome-wide array containing nearly 2,500 bacterial artificial chromosome (BAC) clones with a resolution at about 1.4 Mb was used to illustrate the detection of copy number alterations (CNAs) in various breast cancer cell lines [22]. Recently, a separate study using an array of 422 genomic loci detected frequent alterations at 1, 6, 7p, 9, 11q, 12q, 17, 20q and 22q in archival breast cancer specimens [23]. cDNA arrays have also detected DNA copy changes of amplicons containing *ERRB2 *on 17q [24-27]. More recently, a cDNA array containing 6,691 mapped human genes was used to explore the relationship between copy number alteration and gene expression changes in breast tumors and cell lines [28]. While large-insert clone megabase-interval CGH arrays and cDNA arrays provide a robust platform for the rapid survey of tumor genomes, valuable information could be overlooked as a result of their limited resolution. It is clear that a more detailed description of breast tumor genomes would require re-examination with a higher-resolution array platform.

Genetic, biochemical and pharmacologic studies of breast cancer have been greatly dependent on several commonly used model breast cancer cell lines: MCF-7, BT-474, SK-BR-3, T-47D, UACC-893, MDA-MB-231 and ZR-75-30. That is, a summation of studies involving at least one of these seven cell lines produces over 13,500 hits on Medline. These cells are known to harbor gross chromosomal aberrations; measuring the precise segmental copy number status across their entire genome may uncover novel discrete changes. In the current study we expanded the use of array CGH to survey the genomes of these breast cancer cells at unprecedented detail with a recently developed whole-genome tiling path array that covered the genome with 32,433 overlapping BAC clones [29]. Analysis at this resolution has led to the identification of novel features in these genomes and to the delineation of segmental genetic alterations that have escaped detection by conventional molecular cytogenetic techniques and previous marker-based or interval array CGH analysis.

## Materials and methods

### Cell line DNA

A panel of seven breast cancer-derived cell line DNA was obtained from the American Type Culture Collection: MCF-7, T-47D, Sk-Br-3, MDA-MB-231, BT-474, UACC-893 and ZR-75-30. Pooled normal female DNA was used as reference for all array CGH experiments (Novagen, Mississauga, ON, Canada). DNA was quantified with a NanoDrop ND-1000 spectrophotometer (NanoDrop Technologies, Wilmington, DE, USA).

### Array CGH

The seven cell lines were assayed for genetic alterations with a whole-genome tiling path BAC array in comparative genomic hybridization experiments. The submegabase-resolution tiling set (SMRT) array contains 32,433 overlapping BAC-derived DNA segments that provide tiling coverage over the human physical genome map. All clones were spotted in triplicate, resulting in 97,299 elements over two sides [29-31]. A detailed protocol is provided in Additional file 1.

### Imaging and analysis

Hybridizations were scanned with an imaging system based on a charge-coupled device (Arrayworx eAuto; Applied Precision, Issaquah, WA, USA) and analyzed with SoftWoRx Tracker Spot Analysis software. Stringent criteria were applied to filter spot intensity data. A standard deviation greater than 0.075 between triplicate spots was deemed unreliable and such spots were therefore excluded from our analysis [29]. Only data points with a ratio of signal intensity to background intensity noise exceeding 15 were used in the analysis.

Custom software (SeeGH) was used to visualize log_2 _ratios of clones with respect to location in the genome [32,33]. Because of the complexity of the genomes of these cell lines with respect to ploidy, we have set thresholds for high-level gains and losses to +0.8 and -0.7, respectively, to limit the number of regions for discussion. This threshold encompasses high-level or multi-copy changes previously reported while excluding the abundant number of low-level or single-copy changes common to these cell lines. The complete data set has been made publicly available for further inquiry. In addition, only those loci containing two or more altered overlapping clones were included in the analysis to reduce false positives, and breakpoints were confirmed with the publicly available aCGH-smooth software [34,35].

### Fluorescence *in situ *hybridization

For fluorescence *in situ *hybridization (FISH) probe synthesis, DNA samples from BAC clones RP11-118L18, RP11-419H8, RP11-813P3 and RP11-790I13 were amplified with a modified ligation-mediated polymerase chain reaction protocol as described previously [31]. Imaging and analysis were performed as described previously [36].

## Results and discussion

### Whole-genome tiling path analysis of segmental alterations

**Figure 1:**
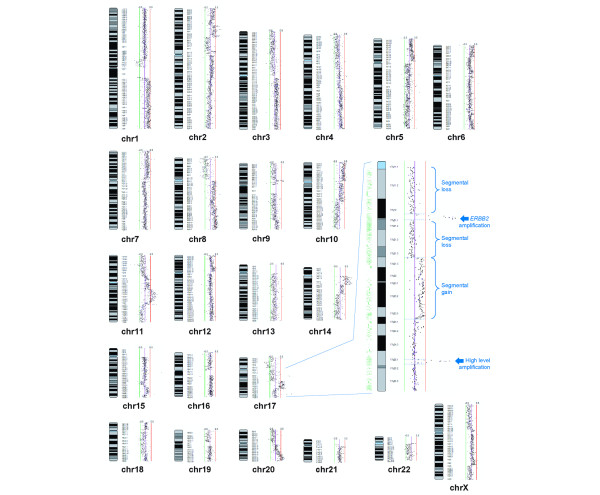
Comprehensive submegabase-resolution tiling set (SMRT) array comparative genomic hybridization profile of cell line UACC-893. Whole-genome SeeGH karyogram UACC-893. Individual data points denote log_2 _ratios plotted to corresponding chromosomal location. Log_2 _± 0.5 scale bars are included for reference. Displacement of data points to the right and left of the centre line represents gain and loss, respectively. The inset shows a magnified view of complex alteration on chromosome 17q.

SMRT array CGH technology provides a tool for assessing genomic aberrations comprehensively in great detail. Comprehensive genomic profiles of segmental gains and losses for seven commonly used breast cancer model cell lines were revealed with this technology. Because of the large amount of data generated, we present the complete genomic profiles and frequency analysis in Additional files 2, 3, 4, 5, 6,7 and 8 (Figure 1). The raw data of the signal intensity ratios of the 97,299 spots for each array CGH experiment have been made publicly available [33] and also deposited at the gene expression omnibus (GEO) database at NCBI, series accession number GSE3106.

Figure 1 demonstrates the details of a tiling path SeeGH karyogram, summarizing SMRT array CGH results for cell line UACC-893. Whole chromosomal arm gains can be seen at 1q, 5p, 7p, 8q and 10p, whereas arm losses are evident at 3p, 4p, 5q, 8p, 13q, 17p, 19p, 20p and Xp. Smaller segmental changes such as the telomeric gained region of 6p or loss at 10q are readily detected. Complex alterations indicating multiple levels of change are denoted by higher-level peaks embedded within a region of change, for example the central region of the 2p arm. The magnified display of 17q demonstrates the identification of a discrete CNA. Beginning at the centromere, we can see two regions of segmental loss separated by a high-copy-number amplicon containing the *ERBB2 *gene. The centromeric breakpoint of this amplicon is located between the overlapping regions of clones RP11-25P3 and RP11-592L16, whereas the telomeric breakpoint is located between clones RP11-686E5 and RP11-259G21. The second region of segmental loss at 17q21.1-q21.31 is followed by a large segmental gain and a second discrete multiple copy amplification at 17q25.1.

To establish detection sensitivity, we first examined previously reported regions of CNA. Our data indicated high-level gains at the c-*MYC *locus in SK-BR-3 and MCF-7 (+2.84 and +1.19 log_2 _ratios, respectively) corresponding to previously reported change in copy number [7,37,38]. Similarly, BT-474, ZR-75-30, UACC 893 and SK-BR-3 are known to harbor a high-level amplification of the *ERBB2 *locus. SMRT array CGH, in addition to detecting the *ERBB2 *locus, revealed several additional discrete changes on the 17q arm in these cell lines. In another example, a previously reported homozygous deletion at 3q13.31, detected by a 10K single nucleotide polymorphism (SNP) array in MCF-7, yielded a log_2 _ratio of -1.2 in our SMRT array CGH analysis [39]. Further comparison of SNP data and SMRT array CGH for cell line BT-474 showed that many of the alterations detected by SMRT array CGH were not clearly delineated or were not detected by the SNP platform (Additional file 9). Although SNP arrays offer the advantage of genotype data, they are only suited to the detection of large-scale changes in copy number. However, the two technologies are clearly complementary because each is designed to address a different question.

Six of the seven cell lines (not MDA-MB-231) were previously profiled for genomic alterations with the use of a 6,691-gene cDNA microarray [28]. Pollack and colleagues showed numerous genomic alterations, both gains and losses, which were correlated with expression patterns on the same array platform. All the CNAs reported were detected by SMRT array CGH, along with the discovery of numerous novel alterations when re-evaluated at tiling path resolution. Known and novel CNAs for the seven genomes are summarized in Table 1. Interestingly, not all CNAs contain annotated genes, which is consistent with the fact that the annotation of coding and non-coding transcripts within the human genome sequence is a continuing process.

### Novel features of the genome of model cell lines

Among the seven cell lines, 75 regions of high-level (multi-copy) segmental gains and 48 regions of multi-copy loss were identified. Because these cell lines serve as model systems for investigating breast cancer biology, a detailed understanding of their genetic alterations is essential to the interpretation of studies with these cell lines. We first describe noteworthy features of the individual genomes and then compare across multiple profiles to identify common alterations.

#### MCF-7 genome

**Figure 2:**
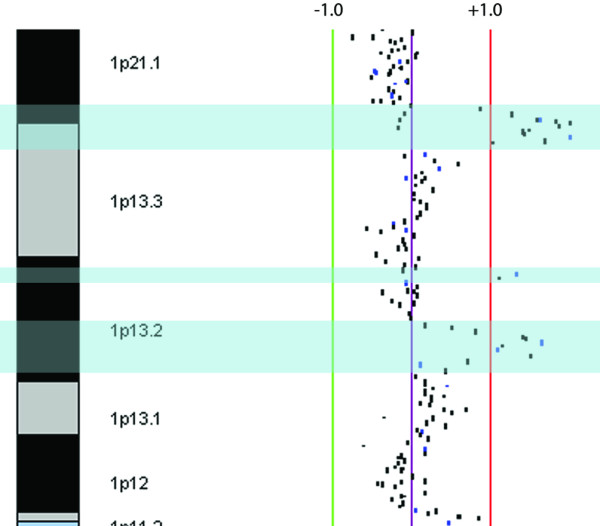
Magnified submegabase-resolution tiling set (SMRT) array comparative genomic hybridization profile of the 1p21.1-p11.1 region in MCF-7. Scale bars labelled ± 1 denote log_2 _ratio scale. Blue highlighted regions indicate the locations of independent amplicons.

The MCF-7 genome harbors 21 high-level CNAs, summarized in Table 1. Remarkably, many of the previously reported regions of genetic alteration split into multiple segments upon tiling resolution analysis. The 1p13 amplification described previously [40] in fact divides into three distinct segments of high-level amplifications: a 1,300 kb segment at 1p13.3, containing only two genes, those encoding arginine N-methyltransferase-6 (*PMRT6*) and netrin G1 (*NTNG1*); a 300 kb segment at 1p13.2, encompassing a single gene, that encoding potassium voltage-gated channel subfamily D member (*KCND3*); and a 1,300 kb region at the centromeric end of 1p13.2, containing 20 genes including *BCAS2*, which has been shown to be amplified and overexpressed in breast cancer cell lines and tumors (Figure 2) [40-42]. Although a loss at 4p15-qter has been reported [14], we observed a 7 Mb loss at 4q34.3-q35.2. The same group also reported an 11p loss; however, our data show that this alteration represents a large 45 Mb segment at 11p15.5-p11.2 and an adjacent but distinct 2 Mb loss at 11p11.2. Similarly, amplifications at the distal end of 15q [13,14] were fine mapped to reveal a 4.9 Mb high-level gain at 15q21.1-q21.3 encompassed by clones RP11-416B20 and 664B9 containing *FGF7*, *CYP19A1 *and *MAPK6*. A lower-level gain was also observed at 15q22.2-qter.

#### BT-474 genome

**Figure 3:**
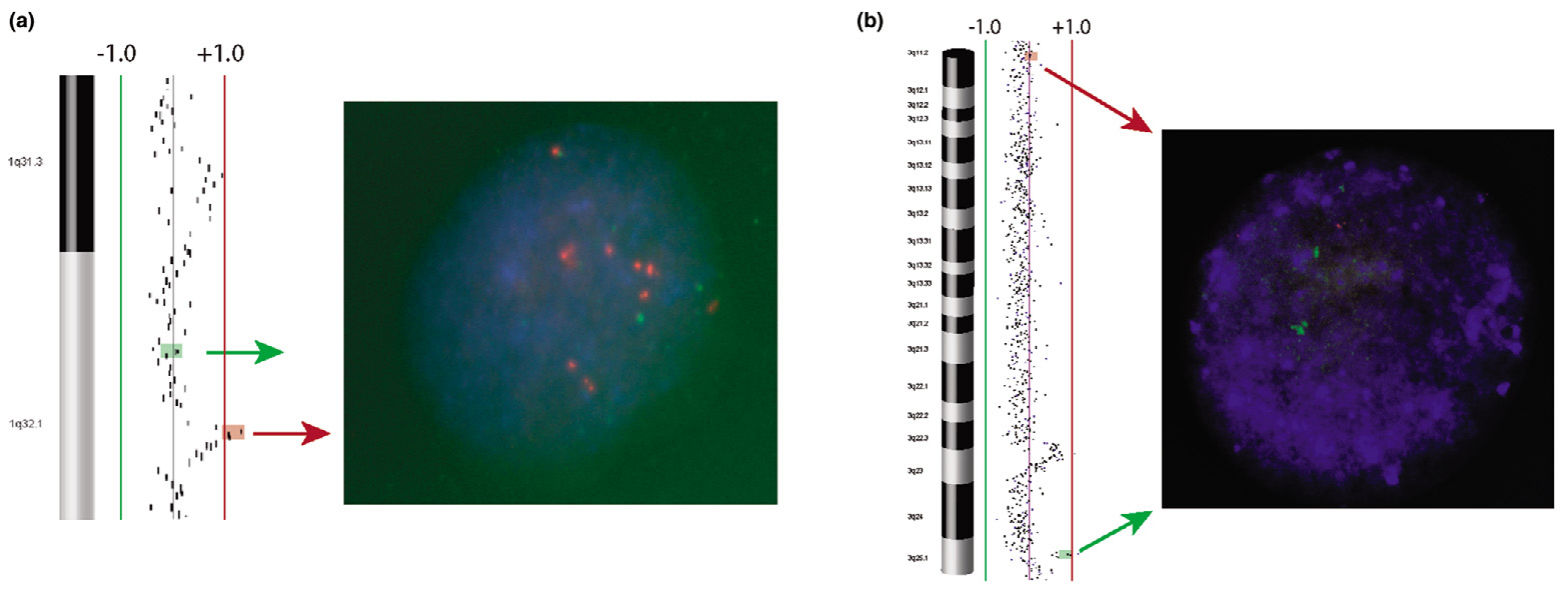
Fluorescence *in situ *hybridization (FISH) analysis in SK-BR-3 and BT-474 cells. **(a) **SK-BR-3 interphase FISH. Clone RP11-118L18, labeled in spectrum green, is located within a 680 kb amplicon at 3q25.1; clone RP11-419H08, labeled in spectrum red, denotes an unchanged site at 3q11.2. **(b) **BT-474 interphase FISH. Clone RP11-813P03, labeled in spectrum red, is located at the peak of a 940 kb amplicon at 1q32.1; clone RP11-790I13, labeled in spectrum green, is located within an adjacent unchanged region.

BT-474 possesses the greatest number of high-level gains and complex alterations and has previously been profiled with the SMRT array CGH platform [29]. In brief, the 1q arm showed multiple rearrangements. A complex aberration at 1q21.2-q25.1 is highlighted by three peaks of high-level gain: 1q21.2-q21.3 (350 kb), 1q22-q23.1 (500 kb) and 1q24.2 (550 kb). In addition, two previously undocumented, distinct regions of gain were identified at 1q31.3 (1,650 kb) and 1q32.1 (950 kb). Figure 3a shows FISH verification of the 1q32.1 amplicon. Although a 1q42-qter gain has been previously reported for BT-474 [14] we observed four separate regions of high-level gain: 1q42.12-q42.13 (500 kb), 1q43 (450 kb), 1q44-q43 (850 kb) and 1q44 (1,700 kb). A 11q13-q14 gain was redefined by SMRT array CGH as a complex high-level amplification at 11q13.1-13.5 (19.8 Mb) containing two distinct and localized high-level peaks at 11q13.1 (700 kb) and 11q13.4 (1,050 kb).

In addition to fine mapping of regions previously reported, several prominent novel alterations were detected: high-level gains at 4q21.1 (2,700 kb), 9p13.3 (2,050 kb), 11q22.1-q22.2 (3,600 kb), 14q11.2-q21.1 (21 Mb) and 14q31.3-q32.12 (3,100 kb). Gains of 20q have been well documented in breast cancer [13,20,23,43]. In BT-474 we observed four distinct segments with increased copy numbers: 20q11.22 (1.3 Mb), 20q13.11-q13.32 (14.8 Mb), 20q13.33 (300 kb) and 20q13.33-tel (1.4 Mb). The gene encoding prefoldin 4 (*PFDN4*) located within 20q13.11-13.32 has been shown to be overexpressed in those cell lines in which it is amplified, including BT-474 [18]. This chromosome arm also harbors regions of loss at 20q11.22 (650 kb) and 20q11.23-13.11 (7,150 kb) that have not previously been reported.

#### ZR-75-30 genome

In total, 11 high-level losses and 13 high-level gains were identified in ZR-75-30. Multiple discrete alterations were observed on chromosome arms frequently implicated in breast cancer, including 1p (four deletions), 8q (eight amplicons) and 17q (seven amplicons and four deletions). Novel segmental losses of varying sizes were detected at 4q21.1 (150 kb), 11q13.5-qter (57.6 Mb) and 21q11.2-q22.11 (16.3 Mb). The discrete high-level amplifications on 8q at 8q11.21 (700 kb), 8q13.3 (500 kb) and 8q22.1 (700 kb) encompassed interesting gene loci such as those for the following: protein kinase DNA-activated catalytic subunit (*PRKDC*), which might have a role in DNA repair and non-homologous DNA end joining; transient receptor potential cation channel A1 (*ANKTM1*), which when overexpressed, affects normal eukaryotic cell growth; and cadherin 17 (*CDH17*), which shares structural features with the cadherin superfamily of calcium-dependent cell–cell adhesion proteins [44-47].

#### UACC 893 genome

High-level gains at 11q13-q14 have been documented in UACC 893 [14]. We also observed this alteration (11q13.3-q14.3, 24.7 Mb); however, an additional discrete high-level gain at 11q22.1 (600 kb) was also discovered, which interrupts a portion of the gene locus for contactin 5 (*CNTN5*), a neural adhesion molecule. A novel gain at 7p21.1 (700 kb) was also detected that encompasses several gene loci, including those for anterior gradient 2 (*AGR2*) and breast cancer membrane protein (*BCMP1*). *AGR2 *has been shown to be positively correlated with estrogen receptor expression and negatively with epidermal growth factor receptor expression in breast cancer tissue [48]. A loss at 16p12.1 (1,400 kb) was also observed.

#### SK-BR-3 genome

Amplifications at 3p22-pter in SK-BR-3 have previously been reported [13,14]. We observed a 400 kb amplification at 3p22.2 as well as two novel regions of high-level amplification at 3q25.1 (700 kb) and 3q22.3-q23 (2,000 kb). Figure 3b shows FISH confirmation of this amplification. Genetic alterations of 8q seem to be complex in SK-BR-3. We observed the three previously reported regions of gain at 8q13.2-q21.13 (10.6 Mb), 8q21.2-q21.3 (6 Mb) and 8q23.2-q24.21 (17 Mb). However, we also identified three distinct amplicons within the 6 Mb region (8q21.2 (300 kb), 8q21.3 (550 kb) and 8q21.3 (500 kb)) and also four distinct high-level peaks within the 17 Mb gain described above: 8q23.3 (750 kb), 8q23.3 (350 kb), 8q24.12 (800 kb) and 8q24.21 (700 kb, contains c-*MYC*). We also observed four regions of deletion not previously reported on 8q: 8q21.3-q22.1 (6 Mb), 8q22.3-q23.1 (4.9 Mb), 8q24.22 (1.6 Mb) and 8q24.23-q24.3 (3.8 Mb). In addition to losses on chromosomes 3 and 8, our analysis has also identified novel regions of loss at 12q23.3-q24.11 (1.4 Mb) and 12q24.21-q24.31 (5.4 Mb) and further delineated a 17q12 gain into two distinct high-level gains at 17q11.1-11.2 (3.2 Mb) and 17q12-21.2 (3.4 Mb). In addition a previously reported gain of 17q24-qter fine mapped to a 1,550 kb amplicon at 17q25.3 [13,14].

#### MDA-MB-231 genome

MDA-MB-231 possessed the fewest number of high-level alterations. Gains at 6p have previously been reported [14,49]; however, two distinct regions of high-level gain were observed within this arm in our analysis: at 6p21.31-21.2 (3.5 Mb) and at 6p21.2-21.1 (3.3 Mb). We also observed a novel 670 kb gain at 7q35. Loss at 9p has also been reported; however, we were able to discern two distinct segmental losses each containing an amplicon [49-51].

#### T-47D genome

T-47D was unique in that it possessed three times as many genomic losses as gains. We observed gains at 18p11.32-p11.32 (350 kb) and 18q21.1 (300 kb) that have not previously been reported [14,38,49,51]. Only five genes reside within the 18q21.1 region: that encoding protein inhibitor of activated STAT2 (*PIAS2*), elongin genes *TCEB3L2 *and *TCEB3L *and hypothetical genes *DKFZP564D1378 *and *HSPC039*.

### Common regions of copy number alteration

Gains at 8q, 17q and 20q are among the most frequently documented alterations in breast cancer. Eight of the nine cell lines (MDA-MB-231 was the exception) showed high-level gains at one or more of these chromosome arms. Multiple alignment of genomic profiles delineated novel minimum altered regions (MARs) common to these cell lines.

Gains at 8q are arguably the most frequently documented alteration in a variety of cancers including breast and prostate cancer [5]. We have highlighted four that were common to multiple cell lines (Additional file 10). First, a discrete 500 kb amplicon at 8q13.3 in ZR-75-30 is also included within the larger alteration at 8q13.33-q21.13 in SK-BR-3. Only one gene resides within this MAR: that encoding transient receptor potential cation channel subfamily A, member 1 (*TRAPA1*). Hyman and coleagues [26] investigated 14 breast cancer cell lines including BT-474, MCF7, SK-BR-3, T47D and ZR-75-30 with a 13K cDNA array identifying four independent genomic amplicons at 8q, including 8q21.11-q21.13, 8q21.3, 8q23.3-q24.14 and 8q24.22. However, the distinct amplicon at 8q13.3 in ZR-75-30 detected by SMRT array CGH was missed in this study. We observed a second larger MAR at 8q21.2-q21.3 common to alterations in MCF7 and SK-BR-3. About 20 genes reside in this 5 Mb region, including those encoding E2F transcription factor, exonuclease GOR and matrix metalloproteinase 16. A third MAR is located at 8q24.12-q24.21 and is common to MCF-7, ZR-75-30 and SK-BR-3, whereas lower-level gains are apparent in BT-474, UACC-893 and MDA-MB-231. Although the genes encoding zinc finger transcription factor (*TRPSI*) and eukaryotic translation initiation factor 3 (*EIF3S3*) are excluded from this MAR (c-*MYC *is included), some of the cell lines possess highly complex gains that extend through a much larger region of the arm and can include the *TRPSI *and *EIF3S3 *loci. Savinainen and colleagues [37] reported 41 copies of *TRPS1 *and 21 copies of *EIF3S3 *and *MYC *in Sk-Br-3. The fourth and most telomeric MAR, 8q24.3, has boundaries defined by a peak of high-level change within the large complex alteration 8q22.2-q24.3 found in ZR-75-30. MCF-7, BT-474 and UACC-893 share low-level gains within this region of about 10 genes.

**Figure 4:**
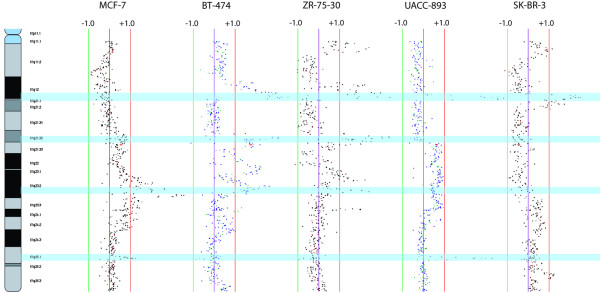
17q SMRT aCGH profile of five cell lines sharing multiple minimum altered regions. Scale bars labelled ± 1 denote log_2 _ratio scale. Blue highlighted regions indicate the locations of MARs.

Chromosome 17q gains have been well documented in both breast cancer cell lines and clinical cases [14,15,21,39,50,52]. Re-examination of this chromosome arm at tiling resolution suggests that the 17q amplification is complex and involves multiple but distinct regions (Fig. 4). First we identified a common high-level gain at 17q25.1 containing a narrow MAR of 760 kb bounded by BAC clones RP11-76G4 and RP11-552F3. The genes encoding RECQ protein-like 5 (*RECQL5*), H3 histone family 3B (*H3F3B*) and growth factor receptor-bound protein 2 (*GRB2*) reside within this gene-rich region, with GRB2 shown to interact with epidermal growth factor receptor (EGFR) [53]. Second, at 17q23, two separate amplicons in MCF-7 and one large amplicon in BT-474 have been described previously, although it is unclear whether these amplicons are overlapping and harbor the same candidate oncogene [25,54]. Our data show the presence of a large complex alteration in MCF-7 at 17q21.32-q24.3 with a high-level amplification at 17q23.2. BT-474 contained two regions of complex alterations at 17q21.32-q23.2 comprising three distinct high-level peaks as well as a single peak at 17q24.1-q24.3 with a single peak. Similarly, three regions of high-level gains were observed in ZR-75-30 and one large region of lower-level gain in UACC-893. Interestingly, our alignment revealed that the high-level peaks involving the 17q23.2 region in MCF-7, BT-474 and UACC893 do overlap, defining a 800 kb MAR from RP11-50F16 to RP11-653P10 containing candidate genes *RPS6KB1*, *LOC51136*, *FLJ22087*, *CA4*, *NY-REN-60*, *APPBP2 *and *PPM1D*.

Another striking feature identified through our tiling resolution scan of 17q is the overlapping amplicons at 17q21.32-q21.33 present in BT-474 and ZR-75-30. The 600 kb MAR from RP11-71G24 to RP11-600O7 harbors the *HOXB *family (*HOXB1 *to *HOXB9)*. Previously described by Hyman and colleagues [26], this amplicon is shown to be present in 10.2% of primary breast cancers, suggesting the involvement of developmental genes in breast cancer pathogenesis (Fig. 4).

Chromosome arm 20q has been shown to be frequently amplified in breast cancer, and amplification of 20q13 is associated with aggressive tumor phenotype, disease recurrence and reduced duration of survival [20]. We identified multiple copy number alterations within this cytoband and defined distinct minimal regions of alteration (Additional file 11). The detection of a 1.5 Mb MAR at 20q13.2 in MCF-7, BT-474 and SK-BR-3 (RP11-20J8 to RP11-346B3) containing the genes encoding zinc finger protein 217 (*ZNF217*), breast cancer-amplified sequence 1 (*BCAS1*), cytochrome P450 24A1 (*CYP24A1*), prefolding 4 (*PFDN4*) and docking protein 5 (*DOK5*) is consistent with previous CGH studies that identified amplification of this region in breast cancer [18,19]. Similarly, we identified a MAR at 20q13.31 from RP11-44A6 to RP11-671P16, containing the gene encoding bone morphogenic protein 7 (*BMP7*), *SPO11 *and the gene encoding RNA export 1 (*RAE1*), corresponding to a previous report in MCF-7 and BT-474 [43]. A large 1.5 Mb amplification at 20q13.12 has also been reported in MCF-7 and BT-474 [43]. Our analysis identified an amplification at 20q13.12-q13.13 common to MCF-7, BT-474 and SK-BR-3. This spanned BAC clones RP11-702E3 to RP11-637D22 defining a narrow 680 kb MAR implicating the genes encoding protein kinase C-binding protein (*PRKCBP1*) and nuclear receptor coactivator (*NCOA3*) as potential oncogenes relevant to breast cancer.

### EGFR (ERBB1) and associated pathways

The EGFR and associated pathways have an important role in several aspects of mammalian cell growth such as cell survival, proliferation and differentiation [55,56]. The receptor family is composed of four type-1 tyrosine kinases (ERBB1 to ERBB4) that dimerize after stimulation by ligand and initiate downstream signaling. Receptor ligand recognition is redundant to some extent, and affinity varies. Although ERBB2 has no known ligand, it becomes activated after heterodimerization with other ERBB family members, the most preferred and potent combination being with ERBB1, whereas the ERBB3 homodimer remains inactive [57].

The redundancy of this pathway suggests its importance as cells have invested in the mechanisms to make this regulatory pathway fail safe. We have investigated genomic loci for about 60 genes implicated in this pathway (Table 2) [56]. Overall, gains were 2.4 times more frequent than losses, and all cell lines contained at least three loci of change. Our data revealed that, as expected, the *ERBB2 *locus is highly amplified in four cell lines (UACC-893, ZR-75-30, BT-474 and SK-BR-3), and overexpression has been shown in two of them [9]. Although amplification of EGFR-interacting genes *RECQL5*, *H3F3B *and *GRB2 *has been described above, other frequently altered loci include c-*MYC*, *LIMK1*, *PRCKA*, *CHN2*, *ERBB2*, *PYK2*, *MAP2K3*, *MAP2K3 *and *PLG1*. Interestingly, T-47D and the two *ERBB2*-overexpressing lines, BT-474 and SK-BR-3, share amplifications at five gene loci: *MAP2K6*, *CHN2*, *PRKCA*, *LIMK1 *and c-*MYC*.

## Conclusion

We examined the genomes of seven commonly used breast cancer cell models in unprecedented detail for segmental copy number status, cataloging the boundaries of gains and losses throughout these genomes. In addition, we demonstrated that copy number alteration of multiple genetic loci involved in the EGF family of pathways is common in these cell lines, which suggests that disruption of this frequently dysregulated pathway in breast cancer may occur at several points in the signaling cascade and that several disruptions may occur in combination.

Furthermore, because these cell lines serve as models for studying the molecular biology of breast cancer, it is essential to take into account the potential influence of genetic alterations when interpreting biological data. For example, using these lines to study the EGF family of pathways, multiple endogenous genetic alterations may have a role in biochemical and biological observations. Our work provides a comprehensive list of high-level segmental gains and losses for each genome, providing a database of copy number alterations as a resource for breast cancer research with these cell lines.

## Abbreviations

BAC = bacterial artificial chromosome; CGH = comparative genomic hybridization; CNA = copy number alteration; EGFR = epidermal growth factor receptor; FISH = fluorescence *in situ *hybridization; kb = kilobases; MAR = minimum altered region; Mb = megabases; SMRT = submegabase-resolution tiling set; SNP = single nucleotide polymorphism.

## Competing interests

The authors declare that they have no competing interests.

## Authors' contributions

AS performed the array CGH experiments, data analysis and drafted the manuscript. WLL is the Principal Investigator. Both authors participated in the development of concepts and framework for the manuscript, the generation of figures, multiple rounds of text editing, and fact checking. Both authors read and approved the final manuscript.

## Supplementary Material

Additional File 1 A Microsoft Word file containing the detailed SMRT array CGH protocol. Click here for file

Additional File 2 A PDF file containing a BT474 Karyogram. Click here for file

Additional File 3 A PDF file containing a MCF7 Karyogram. Click here for file

Additional File 4 A PDF file containing a T47D Karyogram. Click here for file

Additional File 5 A PDF file containing a SKBR3 Karyogram. Click here for file

Additional File 6 A PDF file containing a MDA MB 231 Karyogram. Click here for file

Additional File 7 A PDF file containing a ZR 75 30 Karyogram. Click here for file

Additional File 8 A PDF file containing a frequency plot of 7 cell lines. Click here for file

Additional File 9 A PDF file containing an SNP SMRT aCGH comparison of 4q and 17q in BT474. Click here for file

Additional File 10 A PDF file containing a multiple alignment of 8q. Click here for file

Additional File 11 A PDF file containing a multiple alignment of 20q. Click here for file

## Figures and Tables

**Table 1:** High-level alterations detected by array CGH

Cell line	Locus	Start clone	End clone	Size (kb)		Locus	Start clone	End clone	Size (kb)
**MCF7**	**Amplifications**					**Deletions**			
	1p13.3	N0451I14	N0228E23	1,290		3q13.31	N0747H24	N0362H11	700
	1p13.2	N0099M15	N0795O09	288		4q34.3-35.2	N0442N05	N0746B09	7,100
	1p13.2	N0626F04	N0517B05	1,330		6q25.2-27	M2007C03	M2258B24	14,400
	3p14.2-14.1	N0669F02	N0589G04	3,180		8p arm	p	p	p
	8q21.2-q24.21	N0133G02	N0315E09	43,300		11p15.5-11.2	N0412M16	M2326E01	45,300
	15q21.1-21.3	N0416B20	N0664B09	4,930		11p11.2	N0070A09	M2326E01	1,940
	17q23.2-24.3	N0716B04	N0203A19	113,200		11q11-q12.1	N0010E21	F0627I09	4,300
*Peak:*	*17q23.2*	*N0760B22*	*N0433B24*	*4,900*		11q14.2-23.3	N0282G16	N0004N09	30,000
	17q25.1	N0076G04	N0552F03	766		11q23.3-25(tel)	N0196E01	N0715D10	15,400
	20q12	N0385G02	N0476P15	1,790		13q14.2-34(tel)	N0155D15	M2323L19	66,900
	20q13.12-20q13.33 (tel)	N0272C13	N0476I15	17,500					
*Peaks:*	*20q13.12-20q13.13 ****A***	*N0702E03*	*N0730O20*	*1,790*					
	*20q13.13 ****B***	*N0711M06*	*F0592G15*	*309*					
	*20q13.2 ****C***	*N0020J08*	*N0346B03*	*1,450*					
	*20q13.31* ***D***	*N0044A06*	*N0671P16*	*411*					
	21q22.13-22.3(tel)	N0094J12	N0457P07	8,450					
**BT474**	**Amplifications**					**Amplifications**			
	1q21.2-q25.1					15q11.2 -q12	N0607H20	N0208F21	5,030
*Peaks:*	*1q21.2-q21.3* ***A***	*N0035F14*	*N0714N02*	*366*	*Peaks:*	*15q11.2 ****A***	*N0552D03*	*M2200G17*	*949*
	*1q22-q23.1* ***B***	*N0647N20*	*N0740J19*	*510*		*15q11.2 ****B***	*N0484P15*	*N0710L06*	*624*
	*1q24.2* ***C***	*N0137J06*	*N0616K15*	*540*		17q12-21.2	N0196P12	N0278E15	4,090
	**1q31.3**	***N0662E13***	***N0141E20***	***1,661***		17q21.32-23.2	N0771D19	M2014K24	13,800
	**1q32.1**	**N0783D13**	**N0617D19**	**936**	*Peaks:*	*17q21.32-q21.33* ***A***	*N0071G24*	*N0607H13*	*2,500*
	1q42.12-q42.13	M2185P06	M2016D17	*500*		*17q22-23.2 ****B***	*N0515J20*	*N0473G17*	*2,180*
	1q43	N0236L13	N0614N14	449		*17q23.2 ****C***	*N0399O18*	*N0767P09*	*1,170*
	1q44-q43	N0440F10	N0794A13	865		17q24.1-24.3	N0583F02	N0693H11	5,300
	1q44	N0778E23	N0071K05	1,720	*Peak:*	*17q24.1*	*N0583F02*	*N0394K10*	*482*
	4p16.1-15.33	N0270I03	N0652B07	1,210		19p13.2-13.12	N0295M02	N0441D06	6,310
	**4q21.1**	**N0598G02**	**N0772N01**	**2,700**		20p12.1	N0134G22	N0022E15	*800*
	**9p13.3**	**N0069E18**	**N0795P12**	**2,060**		20q11.22	N0601G07	N0552G16	1,300
	**9q33.1-34.13**	**M2248M11**	**N0738I14**	**12,600**		20q13.11-13.32	N0809G24	N0261P09	14,800
	11q13.1-13.5	N0813P09	N0360N22	19,800		20q13.33	N0648D07	N0694I10	305
*Peaks:*	*11q13.1 ****A***	*N0029K11*	*N0804F01*	*704*		20q13.33-tel	N0305P22	N0134L13	1,380
	*11q13.4 ****B***	*N0093M11*	*N0598G03*	*1,030*		**Deletions**			
	**11q22.1-22.2**	**N0795D03**	**N0347H03**	**3,560**		5q14.1-14.3	N0129E04	N0291O24	5,390
	**14q11.2-q21.1**	**N0597A11**	**N0254B15**	**21,230**		**6q24.1**	**N0709J21**	**M22024J17**	**3,200**
	**14q31.3-32.12**	**N0771O08**	**N0325L17**	**3,080**		**20q11.22**	**N0171G22**	**N0774C15**	**670**
						**20p11.23-13.11**	**N0712N14**	**N0464F07**	**7,140**
**ZR 75 30**	**Amplifications**					**Amplifications**			
	8q11.21	N0569I08	N0513O13	682		17q22-23.2	N0349F01	N0153J08	5,600
	8q13.3	N0367C12	N0634L17	538	*Peaks:*	*17q23.2 ****A***	*N0159D12*	*M2023J07*	*298*
	8q21.2	N0319A24	N0317J10	1,350		*17q23.2 ****B***	*N0381A05*	*N0634F05*	*3,560*
	8q21.3	N0565H20	N0102D03	264		17q23.3-24.1	N0614F05	N0712A10	481
	8q22.1	N0381I07	N0103P22	729		**Deletions**			
	8q22.2-24.3	N0281D17	N0620H01	45,600		**1p36.33-35.3**	**N0206L10**	**N0758C04**	**26,700**
*Peaks:*	*8q23.1* ***A***	*N0078J05*	*N0357L07*	*907*		**1p35.2-35.1**	**N0629A12**	**N0068H10**	**775**
	*8q24.12 ****B***	*N0760H22*	*N0088J18*	*1,440*		**1p34.3**	**N0452M14**	**N0020P17**	**1,510**
	*8q24.22 ****C***	*N0100J23*	*N0422I20*	*440*		**1p21.2-13.3**	**N0293L15**	**N0813H10**	**10,800**
	*8q24.3 ****D***	*N0662P06*	*F0530P15*	*2,740*		**4q21.1**	**N0184F15**	**N0184J11**	**151**
	17q11.1-11.2	N0260A09	N0147N18	3,050		**11q13.5-25(tel)**	**N0149B15**	**M2270L17**	**57,600**
	17q12	N0600K04	N0560P04	*980*		17q11.2-12	N0082D14	N0104J23	6,020
	17q12	N0722D15	N0062P03	*1,360*		17q21.1-21.32	N0278E15	N0046I22	7,700
	17q12-21.1	N0689B15	N0032H06	490		17q23.33-22	N0368A16	N0379H09	994
	17q21.32-21.33	N0771D19	M2190C10	3,910		17q22	N0466D20	N0515J20	881
						**21q11.2-q22.11**	**N0615H23**	**N0694N16**	***16,300***
**UACC 893**	**Amplifications**					**Deletions**			
	**7p21.1**	**N0116D07**	**N0746H13**	**733**		11p15.1	N0118A14	N0614L12	995
	11q13.3-q14.3	M2011L13	N0613M07	24,700		**16p12.1**	**N0104F06**	**N0674B07**	**1,380**
	**11q22.1**	**N0743I15**	**N0659E10**	**619**		17p arm	p	p	p
	17q12-21.2	N0600J16	N0686E05	1,490					
	17q21.33-24.2	N0095M07	N0068K09	17,700					
	17q25.1	N0076G04	N0449J21	999					
**SKBR3**	**Amplifications**					**Amplifications**			
	3p22.2	N0091E04	N0325M12	432		17q12-21.2	N0062P03	N0606M07	3,390
	**3q25.1**	**N0739J07**	**N0118L18**	**678**		17q25.3	N0305C04	N0781F24	1,550
	**3q22.3-q23**	**N0657M13**	**N0718H02**	**2,000**		20p arm	p	p	p
	3q26.2-q26.31	N0190I05	N0590E04	3,300		20q11.22-11.23	N0454F11	N0254N13	4,510
	7q31.1-q32.3	M2023N18	N0019B03	17,800		20q12-13.32	N0434N22	N0290D09	16,200
	8q13.3-21.13	N0746L20	N0125J17	10,600		**Deletions**			
	8q21.2-21.3	N0509F16	N0561A10	5,950		**8q21.3-22.1**	**N0230C03**	**N0804A07**	**6,040**
*Peaks:*	*8q21.2 ****A***	*N0694L21*	*N0606L16*	*309*		**8q22.3-23.1**	**N0739L19**	**N0025P11**	**4,920**
	*8q21.3* ***B***	*N0129P07*	*N0196C06*	*559*		**8q24.22**	**N0015L05**	**N1147M08**	**1,570**
	*8q21.3 ****C***	*N0627A06*	*N0529J09*	*499*		**8q24.23-24.3**	**N0467B24**	**N0613F12**	**3,810**
	8q23.2-24.21	N0114O08	N0294P07	17,100		10q22.3-25.2	N0635P19	N0257E05	30,900
*Peaks:*	*8q23.3 ****A***	*N0058O03*	*N0531C21*	*746*		**12q23.3-24.11**	**N0061L24**	**M2155D19**	**1,380**
	*8q23.3 ****B***	*N0164M09*	*M2118B16*	*365*		**12q24.21-24.31**	**N0412G23**	**N0138I16**	**5,360**
	*8q24.12 ****C***	*N0389M07*	*N0047A23*	*821*		**17q11.2-12**	**N0634A23**	**N0607B02**	**6,460**
	*8q24.21 ****D***	*N0288B17*	*N0294P07*	*716*		**17q21.2-23.2**	**N0400F19**	**N0767P09**	**18,100**
	10q21.1-22.3	N0195P24	N0506E07	25,500		18q12.2-21.2	N0645I23	N0664P08	20,400
	14q31.3-32.12	N0046B20	N0386D20	7,020		19p13.11	N0715L15	N0723M22	4,710
	17q11.1-11.2	N0458L21	N0193M11	3,170					
**MDA MB 231**	**Amplifications**					**Deletions**			
	**6p21.31-21.2**	**N0479F12**	**N0450J18**	**3,510**		**8q24.13**	**N0346M14**	**N0391A17**	**1,020**
	**6p21.2-21.1**	**N0259H15**	**N0769C16**	**3,290**		9p24.2-22.2	N0654D08	N0460F23	14,500
	**7q35**	**N0703N05**	**N0340G20**	**670**	*Peak:*	*9p22.3-22.2*	*N0141K07*	*N0460F23*	*2,000*
						9p21.3-21.2	N0066P03	N0486D12	5,210
					*Peak:*	*9p21.3*	*N0315I14*	*N0730N17*	*280*
**T47D**	**Amplifications**					**Deletions**			
	**3q26.2-29(tel)**	**N0415B12**	**M2110L16**	**28,700**		**12p13.32-12.3**	**N0312A03**	**N0056F05**	**11,700**
	11q23.3-25(tel)	N0081I13	M2013A02	15,200		**18p11.32-11.32**	**N0619C21**	**N0193E15**	**349**
	**Deletions**					**18q21.1**	**N0699O23**	**N0093N16**	**311**
	8p23.1	N0485I05	M2185F10	1,000		Xq	q	q	q
	8p23.1	N0367E11	N0801I21	704					

High-level peaks within complex alterations are denoted by italics. Alterations not previously characterized are in bold. Clones beginning with N0 or F0 belong to the Roswell Park Cancer Institute libraries 11 and 13 (RP11 and RP13), respectively; those beginning with M belong to the Caltech-D (CTD) library.

**Table 2:** Components of the epidermal growth factor receptor pathway affected by copy number change

Gene	Locus	MCF-7	BT-474	ZR-75-30	UACC-893	Sk-Br-3	MDA-MB-231	T47D
EGF	4q25					+		
TGFA	2p13.3		-			-		
NRG2	5q31.2					+		
NRG1	8p12	--		--	-			
								
EGFR	7p12.3-p12.1		+			+		
ErBB2	17q12		+++	+++	+++	+++		
ErBB3	12q13							
ErBB4	2q33.3-q34							
								
SH3KBP1	Xp22.12	--						
RASA2	3q23		+					+
VAV2	9q34.2	+	+	-				
GRB2	17q25.1	++			+++			
PLCG1	20q12	+++	-				+	+
PLCG2	16q23.2			-		-		+
								
RNTRE	10p14							+
SSH3BP1	10p12.1				+			
SOS1	2p22.1		-	-				
PTK2B	8p21.2	-	-	--	--			
SRC	20q11.23		--			+++	++	
NRAS	1p13.2	+++			-			
CDC42	1p36.12		-					
RAC1	7p22.1					+		
								
RIN1	11q13.2		+					
RAF1	3p25.2	+						+
MAP3K4	6q26	--	-					
MAP3K11	11q13.1	+	+++					
PAK1	11q13.5							+
								
ADAM9	8p11.23							+
ADAM12	10q26.2		---		-			
ADAM17	2p25.1		+					
RAB5a	3p24.3					+		
MAP2K1	15q21	-						
MAP2K7	19p13.2			-				
MAP2K4	17q11.2			--	--			
MAP2K3	17p11.2			-	-	-		
MAP2K6	17q24.3		+++			+		+
PDPK1	16p13.3		+					
CHN2	7p15.1		+		+	++		+
PRKCA	17q24.2	+++	++			+		+
								
ERK1	16p11.2	+						
ERK2	22q11.21					-		
MAPK8	10q11.22							+
MAPK9	5q35.3		-	-				
MAPK14	6p21.31						++	
AKT1	14q32.33	+	+					+
BAD	11q13.1		+++					
LIMK1	7q11.23		+	+++		+++		+
PLD1	3q26.31					+		++
								
RPS6KA3	Xp22.12	-						
FOS	14q24.3	+						
JUN	1p32-p31	-						
TP53	17p13.1		+	--				-
MYC	8q24	+++	++	+++	+	+++		+
ELK1	Xp11.23	-	-		+			
JUN	1p32.1	-						
EGF	4q25					+		

Scores: +, log_2 _ratio +0.4 to +0.7; ++, log_2 _ratio +0.7 to +0.99; +++, log_2 _ratio more than +1.0; -, log_2 _ratio -0.4 to -0.7; --, log_2 _ratio -0.7 to -0.99; ---, log_2 _ratio less than -1.0.
