# Supplementary material for: Comprehensive copy number profiles of breast cancer cell model genomes
Source: Breast Cancer Res. 2006 Jan 3;8(1):R9. doi: 10.1186/bcr1370 (PMC1413994; doi:10.1186/bcr1370)
Supplement: Additional File 1 — A Microsoft Word file containing the detailed SMRT array CGH protocol. [file bcr1370-S1.doc]

**S-1: Detailed SMRT aCGH Protocol**

Random primed (RP) labeling of 400 ng of sample DNA and 400 ng of reference DNA was undertaken with Cyanine-5 (Cy5) and Cyanine-3 (Cy3) respectively. RP reaction: 10 µL Promega Klenow 10X buffer, 7 µg/µL random octomers, 400 ng DNA to a final volume of 33 µL. The reaction was boiled for 10 minutes then quick cooled on ice. To this, 8 µL of dNTP mix (2mM dATP, dGTP, dTTP, 1.2 mM dCTP), 45 units of Klenow (Promega), 8 nmoles of Cy3 were added to the cell lined DNA RP reactions and 8 nmoles of Cy5 were added to the female reference DNA reactions. RP reactions were incubated ~18 hours at 37°C. Labeled sample and reference DNA were combined and purified using ProbeQuant Sephadex G-50 columns according to manufactures specifications (Amersham).  Two hundred µg of Cot-1 DNA (Invitrogen, Burlington, Ontario) was added to the purified sample mixture. After ethanol precipitation the mixture was dissolved in 100 µL of hybridization solution (33 mg DIGeasy, 180 ug sheared sperm DNA and 90 µg yeast tRNA in 90 µL water) , which was then denatured at 85°C for 10 minutes and annealed at 45°C for one hour to block repetitive sequences. Forty-five µL of probe solution was added to each of the two SMRT array slides, a coverslip was added and the array was incubated at 45°C for ~40 hours in a sealed hybridization cassette (Telechem International Inc., CA). Arrays were washed five times for 5 minutes each in 0.1X SSC, 0.1% SDS at room temperature, followed by five rinses with 0.1X SSC at room temperature. Slides were dried by centrifugation, 2 minutes at 800 rcf.
