# Supplementary figures and images for: Comprehensive copy number profiles of breast cancer cell model genomes
Source: Breast Cancer Res. 2006 Jan 3;8(1):R9. doi: 10.1186/bcr1370 (PMC1413994; doi:10.1186/bcr1370)

# S-2: BT474 Karyogram

red and green lines represent +1.0 and -1.0 log2 ratio scale references respectively

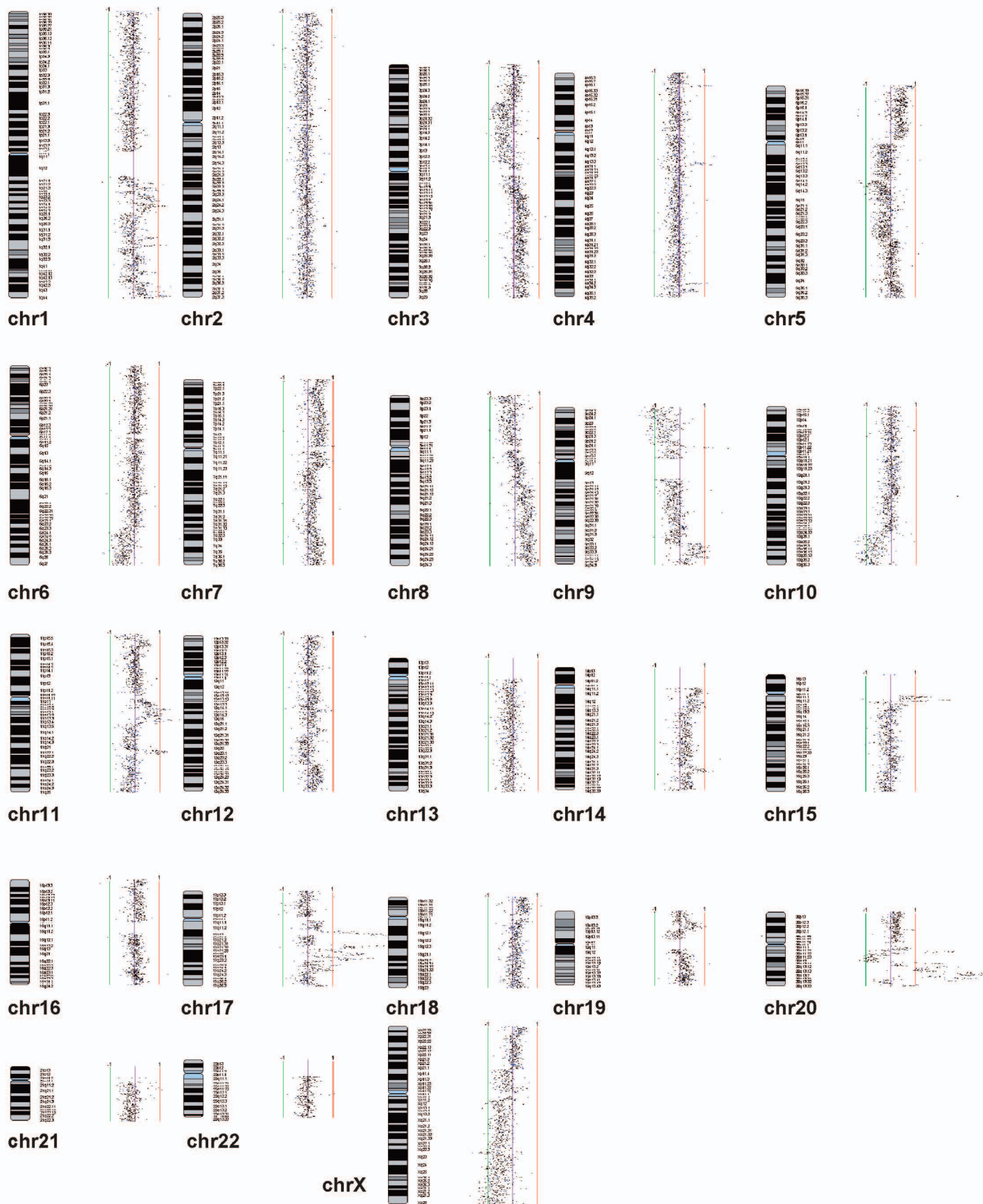

Supplement: Additional File 2 — A PDF file containing a BT474 Karyogram. [file bcr1370-S2.pdf]

# S-3: MCF7 Karyogram

red and green lines represent +1.0 and -1.0 log2 ratio scale references respectively

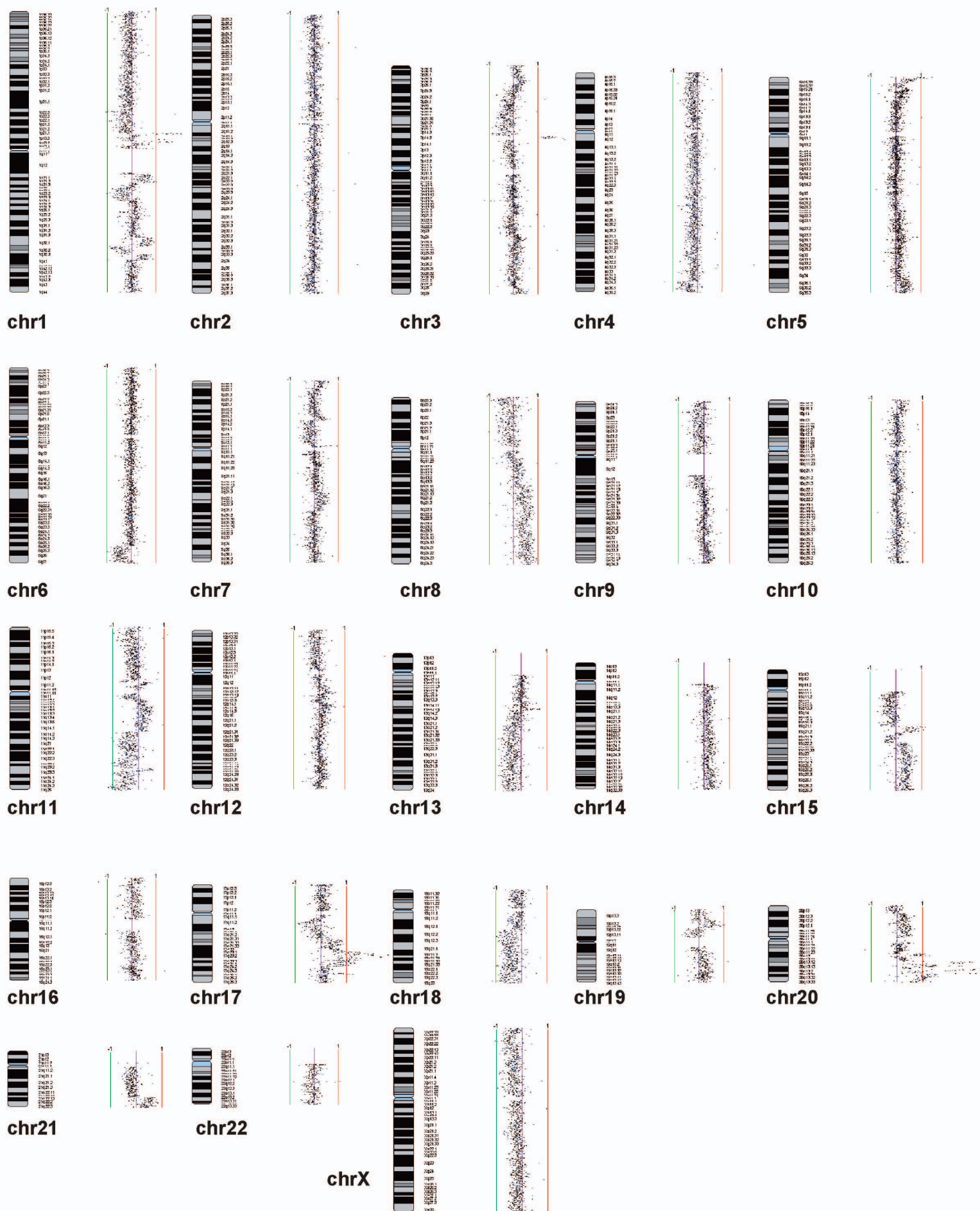

Supplement: Additional File 3 — A PDF file containing a MCF7 Karyogram. [file bcr1370-S3.pdf]

# S-4: T47D Karyogram

red and green lines represent +1.0 and -1.0 log2 ratio scale references respectively

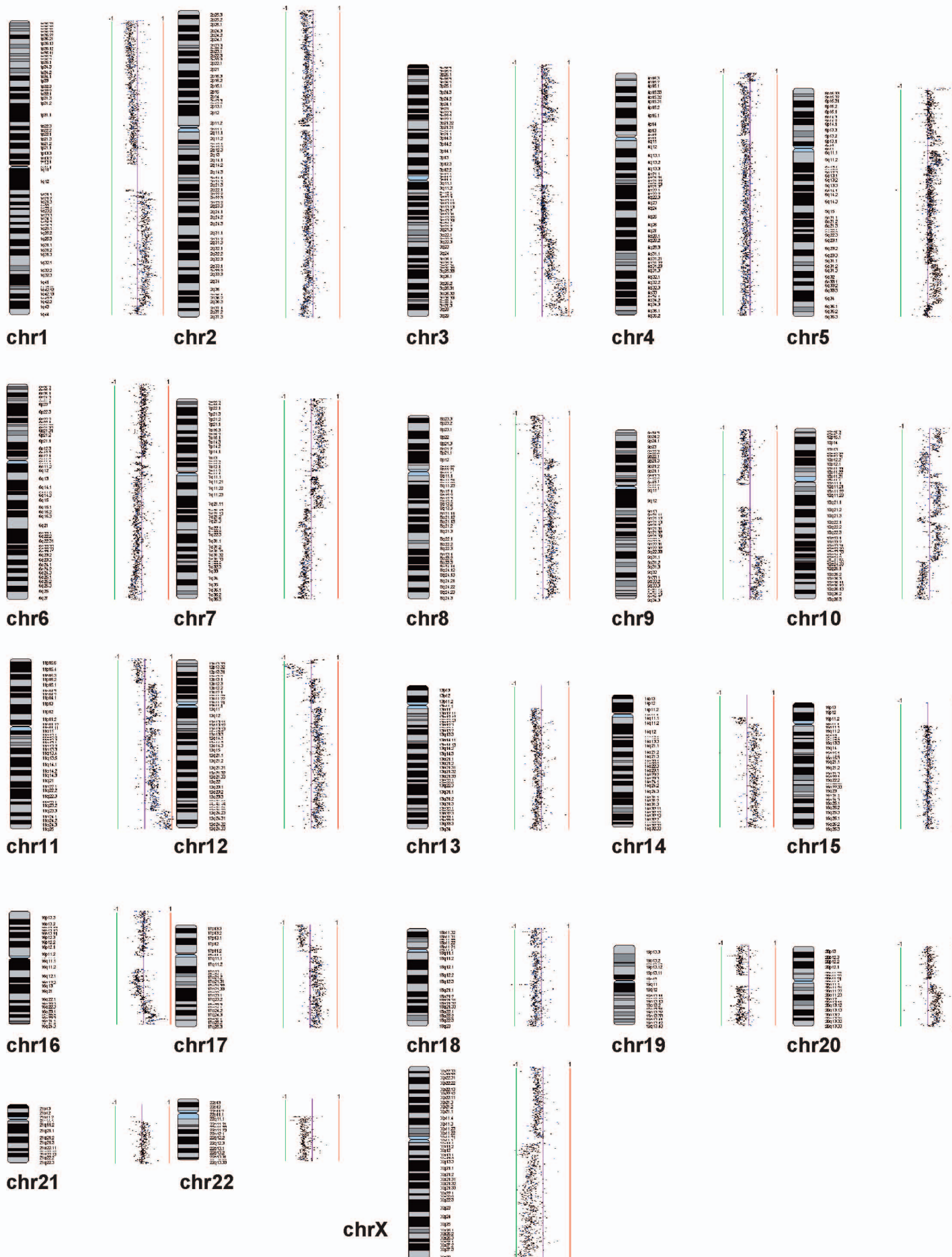

Supplement: Additional File 4 — A PDF file containing a T47D Karyogram. [file bcr1370-S4.pdf]

# S-5: SKBR3 Karyogram

red and green lines represent +1.0 and -1.0 log2 ratio scale references respectively

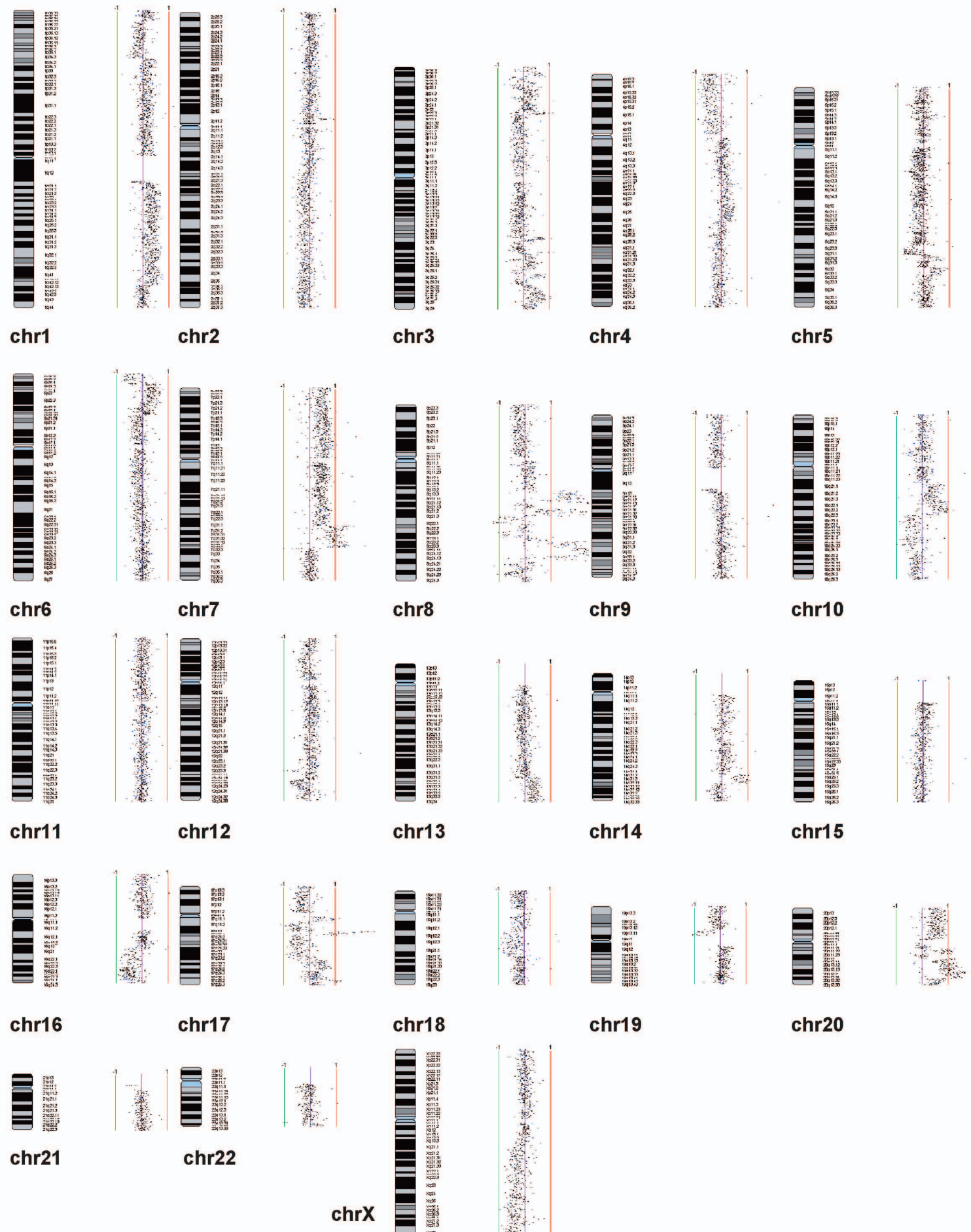

Supplement: Additional File 5 — A PDF file containing a SKBR3 Karyogram. [file bcr1370-S5.pdf]

# S-6: MDA MB 231 Karyogram

red and green lines represent +1.0 and -1.0 log2 ratio scale references respectively

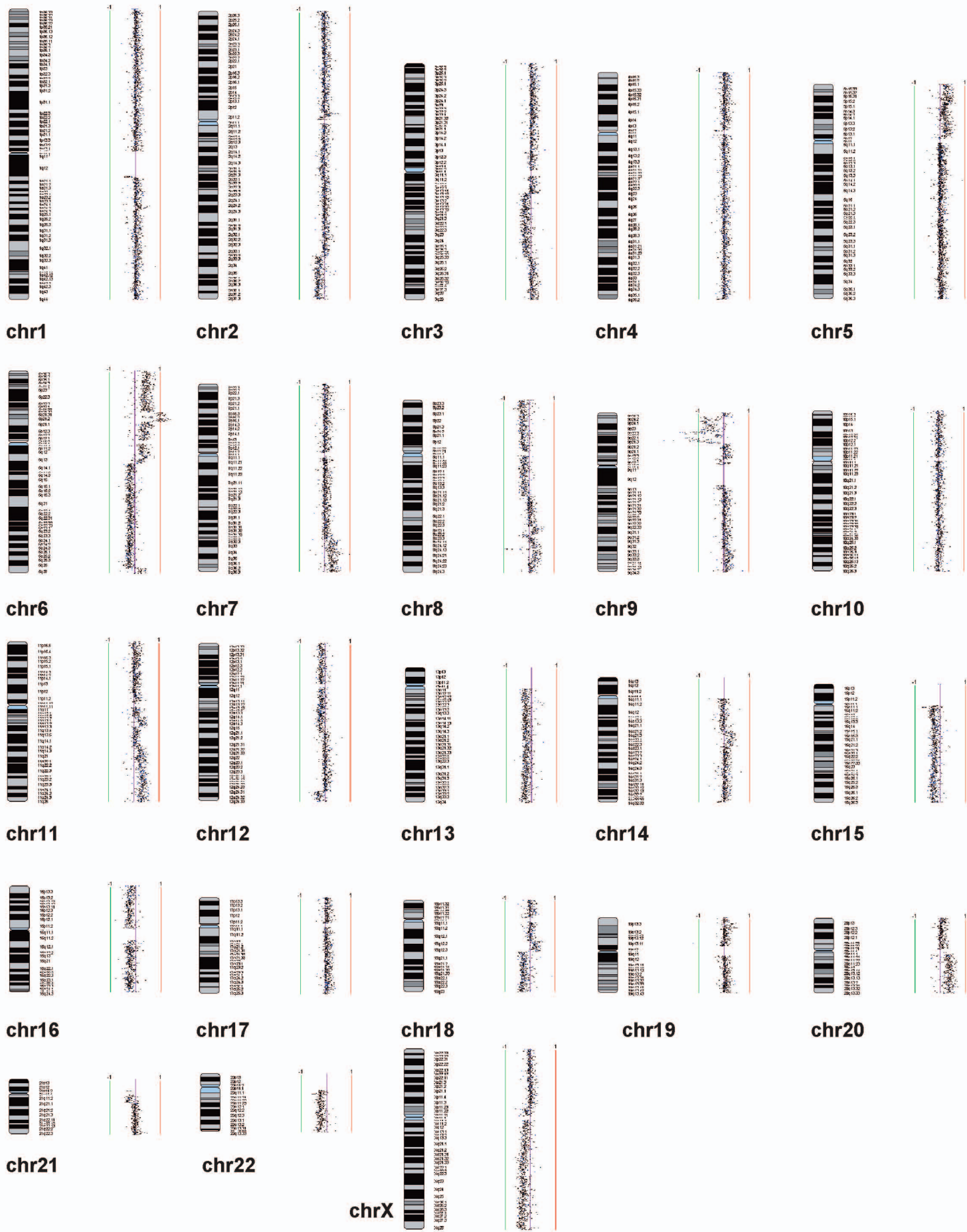

Supplement: Additional File 6 — A PDF file containing a MDA MB 231 Karyogram. [file bcr1370-S6.pdf]

# S-10: 8q Multiple Alignment View

+/-1.0 and +/-0.5 scale bars are included for reference

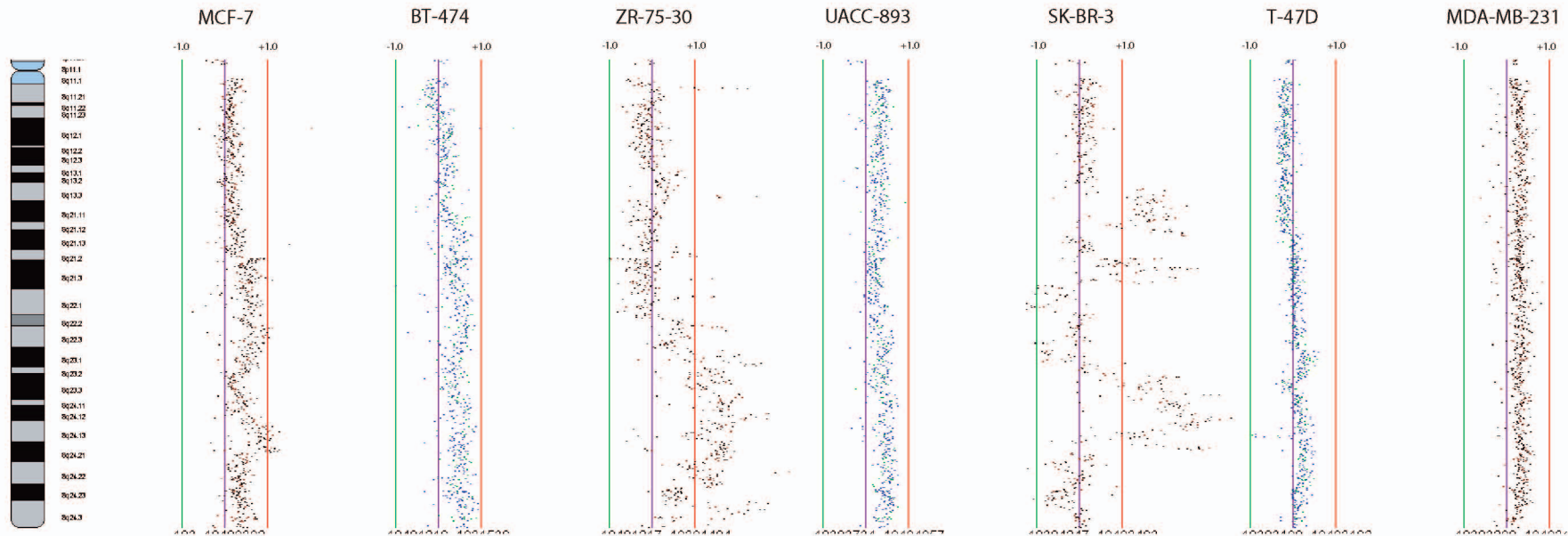

Supplement: Additional File 10 — A PDF file containing a multiple alignment of 8q. [file bcr1370-S10.pdf]

# S-11: 20q Multiple Alignment

+/- 1.0 scale bars are included for reference

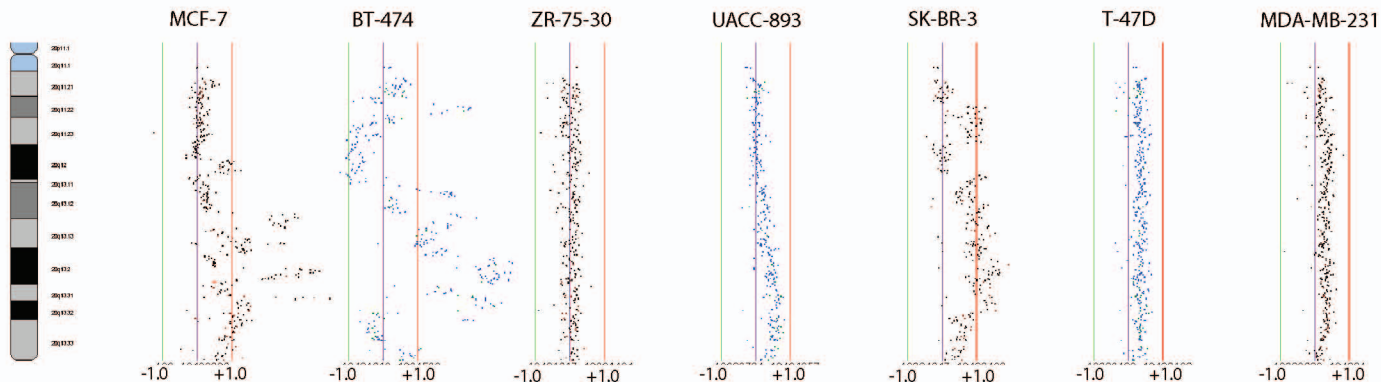

Supplement: Additional File 11 — A PDF file containing a multiple alignment of 20q. [file bcr1370-S11.pdf]
