# Supplementary material for: Comprehensive copy number profiles of breast cancer cell model genomes
Source: Breast Cancer Res. 2006 Jan 3;8(1):R9. doi: 10.1186/bcr1370 (PMC1413994; doi:10.1186/bcr1370)

S-7: ZR 75 30 Karyogram

red and green lines represent +1.0 and -1.0 log2 ratio scale references respectively

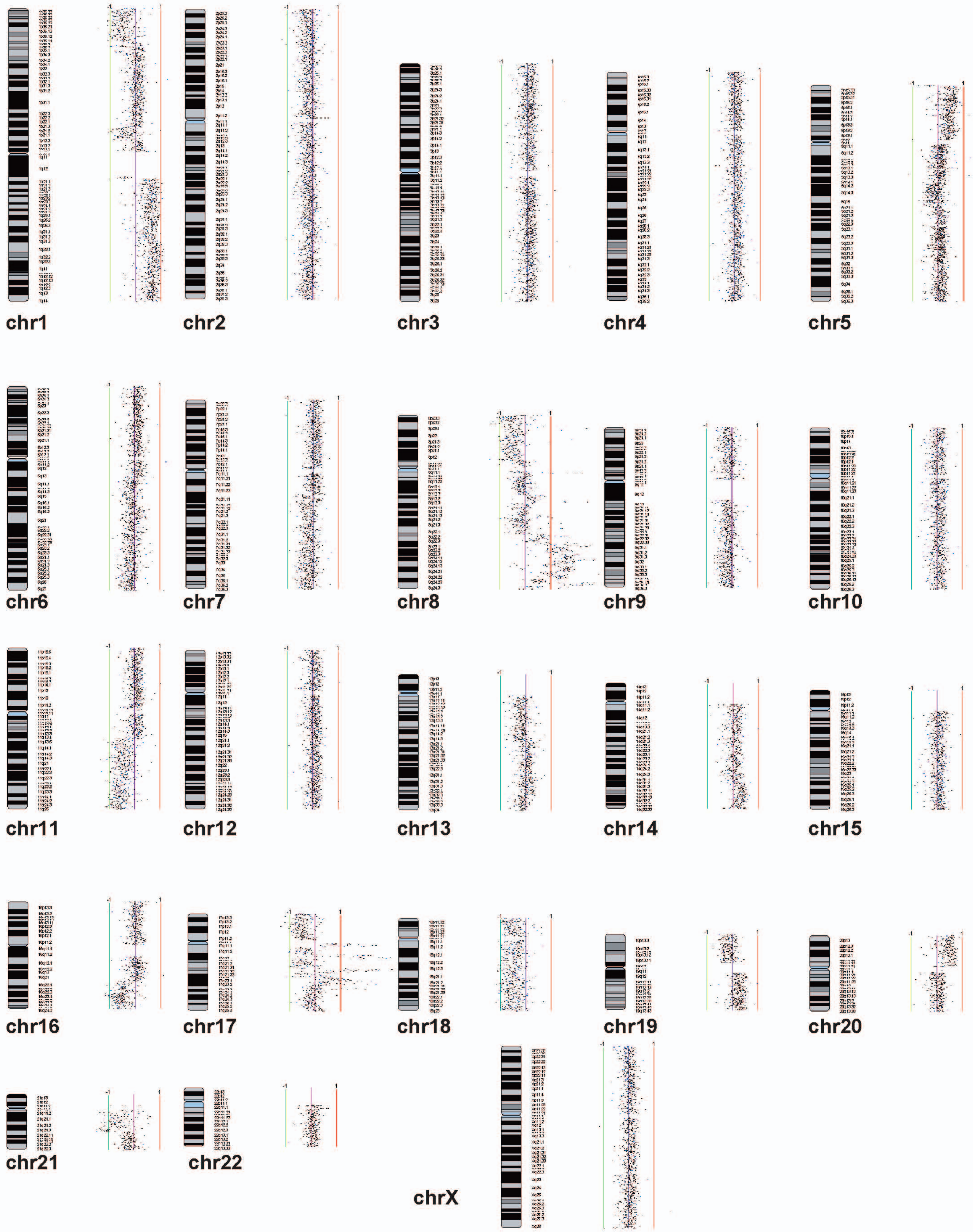

Supplement: Additional File 7 — A PDF file containing a ZR 75 30 Karyogram. [file bcr1370-S7.pdf]
