# Supplementary material for: Comprehensive copy number profiles of breast cancer cell model genomes
Source: Breast Cancer Res. 2006 Jan 3;8(1):R9. doi: 10.1186/bcr1370 (PMC1413994; doi:10.1186/bcr1370)

S-8: Frequency Analysis of Alterations in 7 Cell Lines

Scale bars to the right each chromosome denote +1.0 and +0.5 log2 ratio scale references while those to the left represent -1.0 and -0.5 log2 ratio scale references.

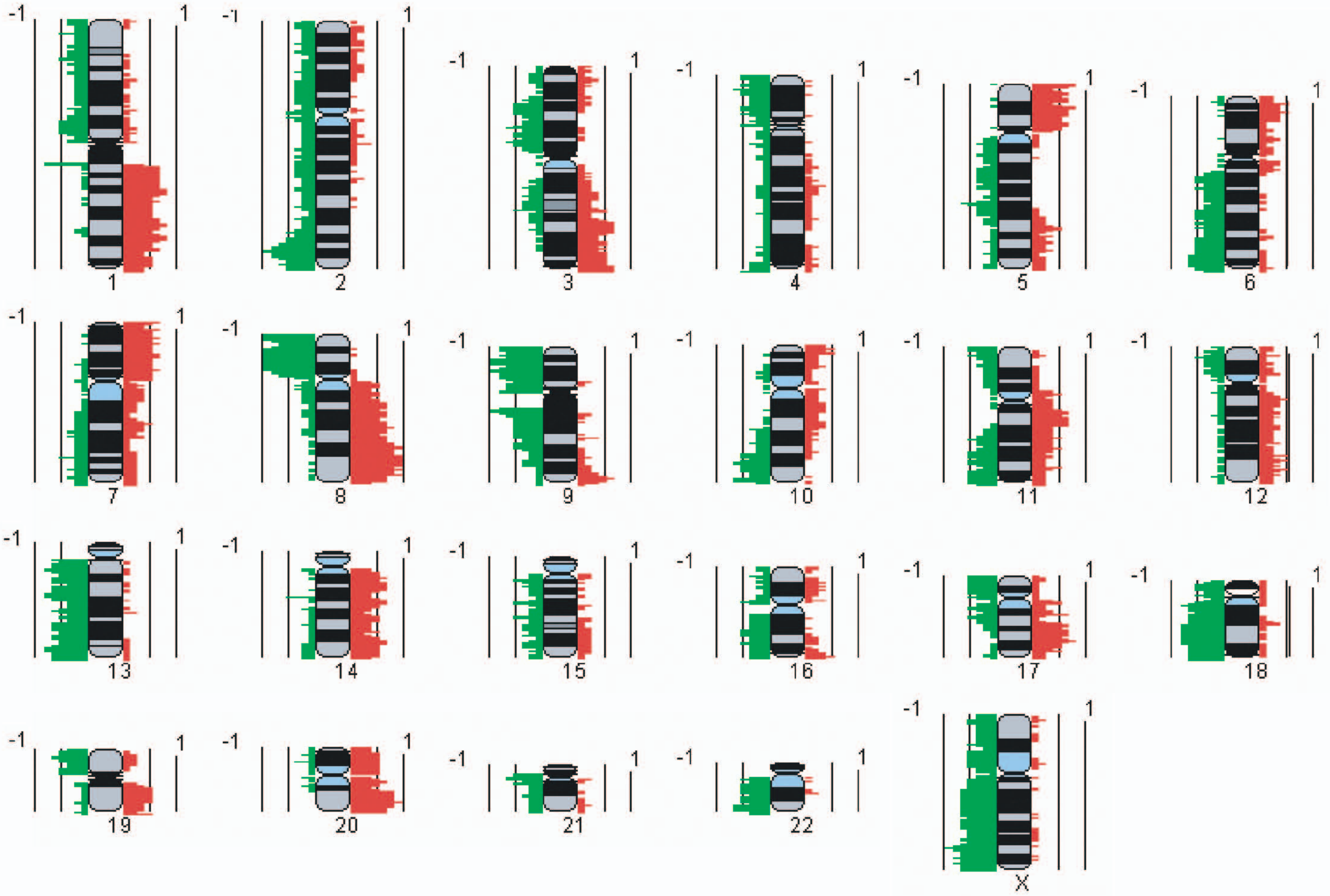

Supplement: Additional File 8 — A PDF file containing a frequency plot of 7 cell lines. [file bcr1370-S8.pdf]
