# Supplementary material for: Comprehensive copy number profiles of breast cancer cell model genomes
Source: Breast Cancer Res. 2006 Jan 3;8(1):R9. doi: 10.1186/bcr1370 (PMC1413994; doi:10.1186/bcr1370)
Supplement: Additional File 9 — A PDF file containing an SNP SMRT aCGH comparison of 4q and 17q in BT474. [file bcr1370-S9.pdf]

S-9: SNP and SMRT array Data Comparison

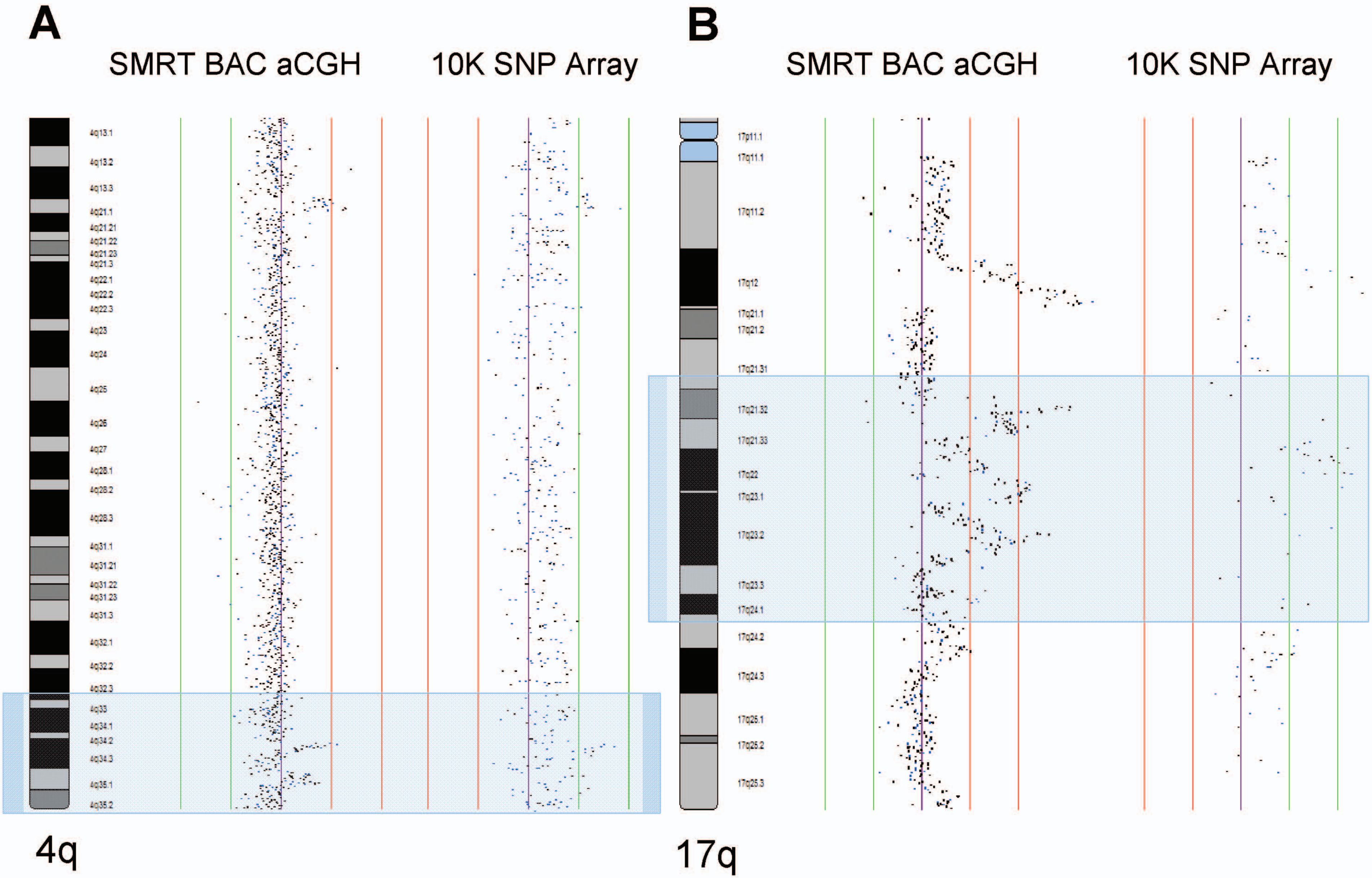

SNP data from Zhao X. *et al* 2004
